# Elemental Selector for High-Density Memory Integration

**DOI:** 10.1007/s40820-026-02279-5

**Published:** 2026-07-13

**Authors:** Shaojie Yuan, Pandeng Xuan, Meng Xu, Ming Xu, Xiangshui Miao

**Affiliations:** 1 School of Integrated Circuits, Huazhong University of Science and Technology, Wuhan, 430074 People’s Republic of China; 2 Yangtze Memory Technology Co., Ltd. (YMTC), Wuhan, 430078 People’s Republic of China; 3 Department of Electrical and Electronic Engineering, the University of Hong Kong, Hong Kong, People’s Republic of China

**Keywords:** Phase-change memory, Ovonic threshold switching selector, Elemental selenium, Monatomic chalcogen, Valence alternation pair

## Abstract

Ovonic threshold switching selectors are indispensable for suppressing sneak currents in dense cross-point memories, but most established selector materials still rely on multicomponent chalcogenides with persistent trade-offs in leakage current, reliability, and compositional stability. Recent progress in elemental switching materials is beginning to change this picture. A new study identifies amorphous selenium as a highly effective selector, combining an ultralow leakage current of 4 × 10^–12^ A, an on/off ratio above 10^8^, a drive current density of 21.2 MA cm^–2^, nanosecond-scale switching, and endurance up to 2 × 10^9^ cycles. More importantly, spectroscopy and theory connect these metrics to a charge-triggered mechanism rooted in dense trap pairs in the amorphous network. These states strongly pin the Fermi level in the off-state, while field-induced carrier release near threshold drives abrupt conduction. Beyond introducing a new selector material, this work suggests that monatomic chalcogens may provide a cleaner platform for understanding and engineering threshold switching, with fewer complications from phase segregation, cation migration, and chemical overdesign.

## Highlights

- Amorphous elemental selenium delivers ultralow leakage, high selectivity, fast switching, and long endurance as an ovonic threshold switching selector.
- Dense trap pairs in amorphous selenium pin the Fermi level in the off-state, whereas field-driven carrier release and avalanche multiplication trigger abrupt turn-on.
- Recent progress in pure Te and pure Se suggests that monatomic switching materials may provide a scalable alternative to compositionally complex selectors.

## Graphical abstract

**Figure:**
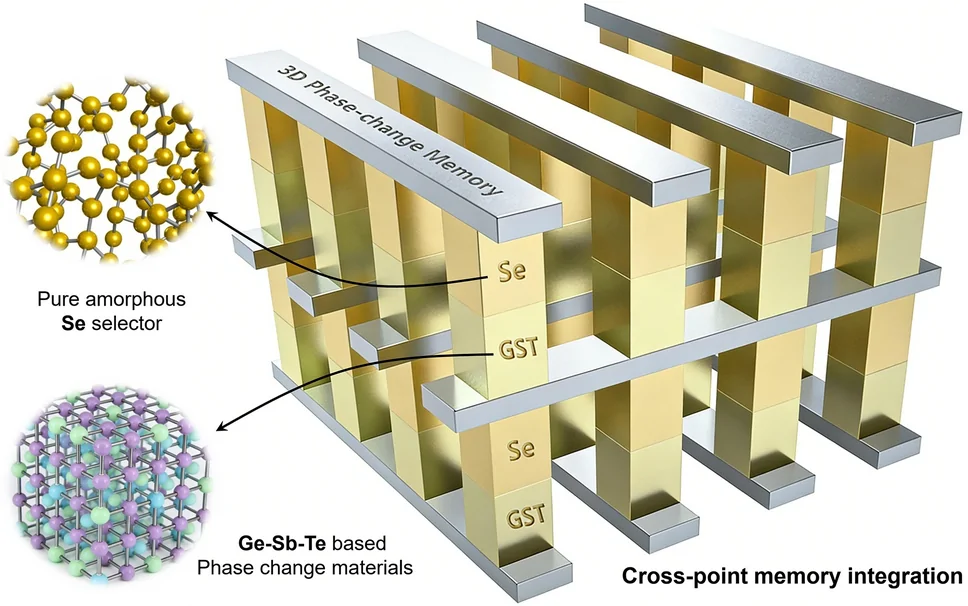

## Introduction

Three-dimensional cross-point memory remains one of the most promising approaches for dense, energy-efficient data storage, with its scalability heavily reliant on selector performance. In these architectures, selectors must block leakage through unselected cells while enabling fast, reversible switching in selected paths. This requirement has positioned ovonic threshold switching (OTS) materials as a core focus of memory research. However, most state-of-the-art OTS materials rely on compositionally complex chalcogenide glasses, whose inherent complexity brings notable limitations [1]. Traditional optimization strategies involve alloying S-, Se-, or Te-based glasses with elements like Ge, Si, In, or As. While such tuning can widen processing windows or stabilize the amorphous phase, it also introduces critical drawbacks: structural aging, elemental segregation, and rising leakage during cyclic operation [1]. This has left the field at an impasse: selector design has grown chemically elaborate, without a corresponding gain in controllability. Accordingly, research attention has shifted to a foundational question: Can threshold switching be realized, and even optimized, in materials with far simpler chemistry?

The first definitive answer came from pure tellurium (Te). Early work demonstrated that elemental Te functions as a volatile switch, eliminating the phase segregation issues common in multicomponent selectors [2]. This finding challenged the long-held assumption that high selector performance requires carefully engineered alloys. A subsequent study stabilized amorphous Te via rapid on-device quenching, further confirming that elemental chalcogens hold both scientific insight and technological relevance [3]. Against this backdrop, the present advance in selenium (Se) is especially notable because it combines elemental simplicity with robust OTS performance, rather than merely establishing elemental switching itself. Together, these advances repositioned elemental switching from a niche curiosity to a viable design pathway.

**Fig. 1:**
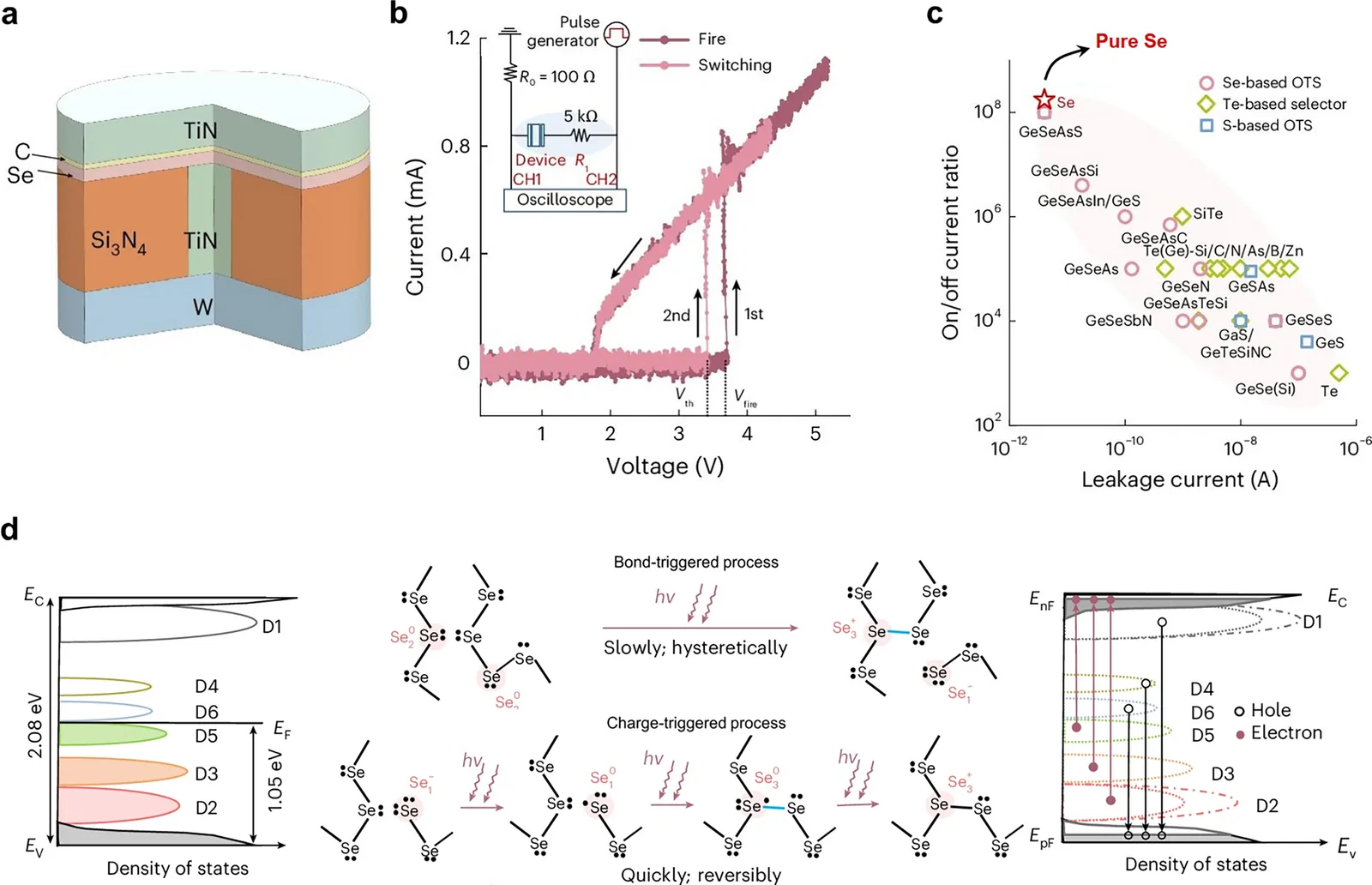
Device structure, representative performance, benchmarking, and switching mechanism of the a-Se OTS selector. **a** Schematic of the confined T-shaped a-Se selector device. **b** Current–voltage characteristics of the a-Se device, showing volatile threshold switching with ultralow leakage and abrupt turn-on. **c** Benchmark comparison of leakage current and on/off current ratio between the a-Se selector and representative S-, Se-, and Te-based selector materials, highlighting the superior leakage–selectivity balance of amorphous Se. **d** Reconstructed density of states of a-Se together with a schematic illustration of the trap-mediated, charge-triggered switching mechanism associated with valence alternation pair states

Building on this momentum, Sun et al*.* [4] developed a confined T-shaped device architecture for a Se-based OTS selector (as shown in Fig. 1a), demonstrating ultralow leakage, high selectivity, fast switching, and robust endurance. The device exhibits an off-state current as low as 4 × 10^–12^ A, an on/off ratio above 10^8^, a drive current density of 21.2 MA cm^−2^, nanosecond-scale switching, and endurance up to 2 × 10^9^ cycles, while also outperforming representative S-, Se-, and Te-based selectors in the leakage–selectivity trade-off (as shown in Fig. 1b, c). The Se selector was further integrated with phase-change memory cells and maintained a functional read margin in 1S1R operation, confirming that this is not merely an isolated materials result, but a practically relevant selector platform for cross-point memory integration. These are not incremental improvements: they place Se devices among the strongest selector candidates reported to date, especially for the key scaling challenge of suppressing off-state leakage without sacrificing fast turn-on.

However, the deeper significance of this work lies in the identification of a charge-triggered switching mechanism in amorphous Se (a-Se) (as shown in Fig. 1d). Rather than framing threshold switching as a purely phenomenological event, the study links the electrical response to the evolution of defect-mediated electronic states under an applied electric field. The a-Se has a wide mobility gap of 2.08 eV, with dense valence alternation pair (VAP)-like trap states within the gap that strongly pin the Fermi level near the bandgap midpoint. This gives the device a large subthreshold activation barrier and ultralow off-state current. These trap states are consistent with local disruptions to ideal twofold coordination. In low-field conditions, the defect-associated states suppress thermally activated carrier transport and stabilize the insulating off-state. Near the threshold field, the dominant process shifts from simple trapping to charge-triggered switching: the increasing electric field excites carriers out of these localized trap states via a fast, reversible charge-triggered process, in contrast to the slower and more hysteretic bond reorganization processes discussed in other switching materials [5], reducing their constraint on carrier transport and driving the system toward band edge conduction. As carrier release continues and the defect landscape evolves, quasi-Fermi levels shift toward the mobility edges, after which impact ionization amplifies the current to enable abrupt turn-on. Notably, the fastest switching event need not require complete detrapping of the full trap population; release of a critical carrier population near threshold can shift quasi-Fermi levels toward the mobility edges and initiate impact ionization-driven current amplification, whereas slower detrapping mainly governs later-stage recovery dynamics. In this framework, VAP-related trap states are not merely passive transport centers inferred from conduction fitting; they provide the microscopic foundation for off-state Fermi-level pinning and serve as the field-responsive carrier reservoir that triggers abrupt turn-on. The conceptual advance is not only the interpretation of these trap states, but also the experimental and theoretical resolution of a defect-mediated switching picture that supports a field-driven charge release mechanism in elemental Se.

This framework gives Se far broader importance than its performance metrics alone. It supports a design principle where selector performance is governed not just by composition, but by how amorphous networks host and reorganize electrically active mid-gap states (MGS). This aligns with recent work showing that MGS in chalcogenide glasses can be tuned via local bonding motifs [6]. It also connects the present VAP-like interpretation to the broader defect physics of lone-pair chalcogenides [7]. Se provides a particularly clear limiting case: a monatomic glass whose intrinsically favorable defect landscape inherently delivers both deep off-state insulation and rapid on-state activation. Rather than relying on multielement alloying to engineer targeted traps, a-Se appears to realize selector-relevant defect physics in a chemically minimal form.

This chemical simplicity also delivers critical reliability benefits. In alloyed selectors, cation-containing additives used to boost amorphous stability or processing tolerance often cause inhomogeneity, filament formation, and parameter drift over time [1]. Elemental Se removes this trade-off at the compositional level and highlights that selector functionality can be achieved without the compositional stabilization strategies (e.g., Si or As doping) historically invoked in many amorphous OTS systems. Although its melting point is relatively low (~ 220 °C), Se also shows excellent glass-forming ability, with a Turnbull parameter of 0.643 confirmed by differential scanning calorimetry [4]. The study further demonstrates that confined Se can recover its amorphous selector state after high-temperature thermal treatment through fast melt-quenching, while retaining low leakage and fast switching. Elemental chalcogen platforms are also relevant from a processing perspective, further supporting their integration interest in dense memory technologies [8]. This moves monatomic Se beyond a mechanistic model system toward a realistic candidate for practical device integration. At the same time, broader implementation will still require careful assessment of thermal budget compatibility, device-to-device variability, process interference, and current compliance strategies during dense array integration.

Looking ahead, Se does not close the book on elemental OTS research; rather, it opens a new avenue for the field. The advances in pure Se and Te show that monatomic chalcogens can emerge as a distinct, high-performance family of selector materials, with value for both device engineering and fundamental studies of threshold switching. This broader trend also echoes earlier progress in monatomic Sb phase-change memory, where compositional simplification was likewise linked to favorable scaling considerations [9]. Three directions now appear especially important: identifying the structural motifs that generate the most effective VAP-related trap distributions, testing how universal the charge-triggered switching mechanism is across monatomic and near-monatomic amorphous glasses, and translating the mechanistic insight gained from Se into rational design rules for binary and ternary selectors. Progress along these directions will require closer correlation among pulsed/transient electrical measurements, bias-dependent spectroscopy, and atomistic modeling, together with tighter control of mid-gap state density, energy depth, spatial distribution, and field response through confinement, thermal history, and interfaces. Overall, the breakthrough of a-Se marks a meaningful shift in OTS research, from composition-heavy empirical optimization toward mechanism-guided and chemically simplified design. Future progress in selector design may thus rely not only on optimizing composition, but also on deliberately tailoring electrically active mid-gap states within amorphous chalcogen networks. For selector engineering, it offers a well-characterized high-performance candidate; for three-dimensional cross-point memory, it provides a scalable route to leakage control and device uniformity. More broadly, such chemically simple selector platforms may also prove useful in other selector-assisted dense memory architectures where leakage suppression, current isolation, and device uniformity remain central challenges.
